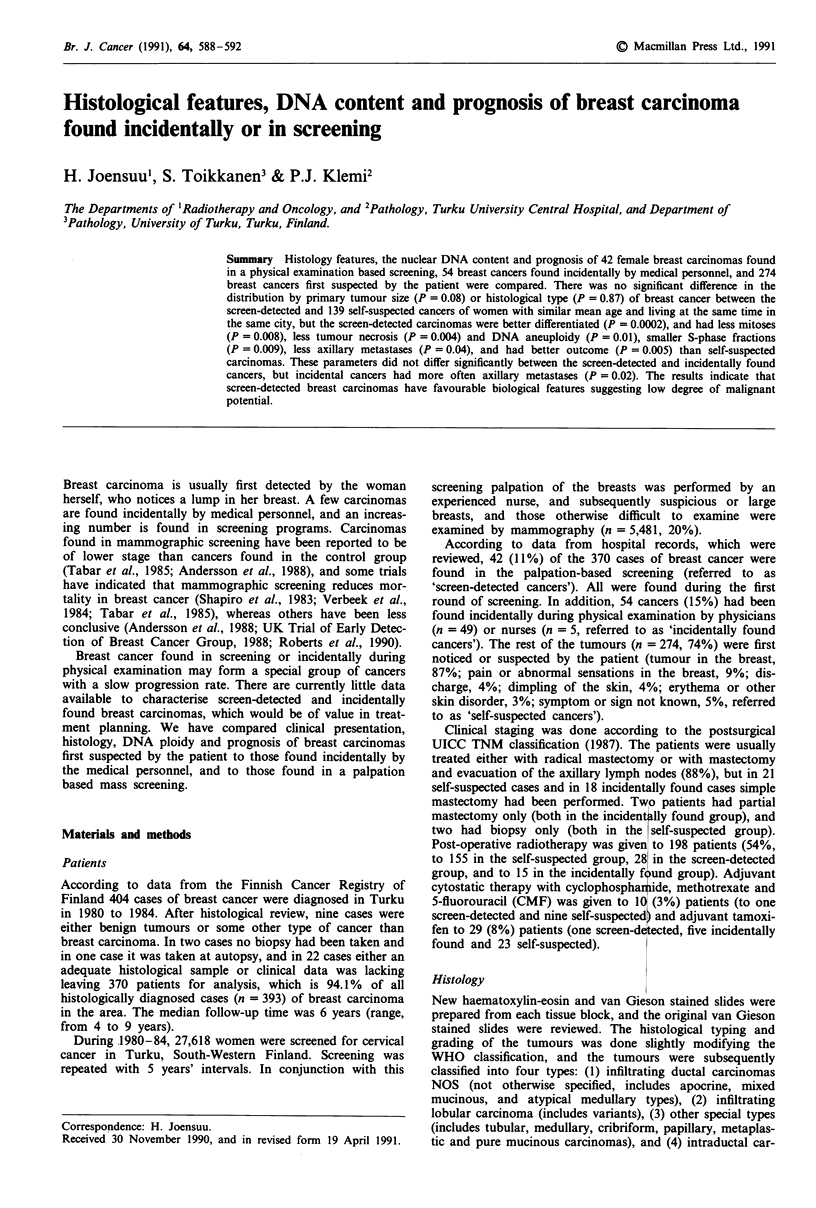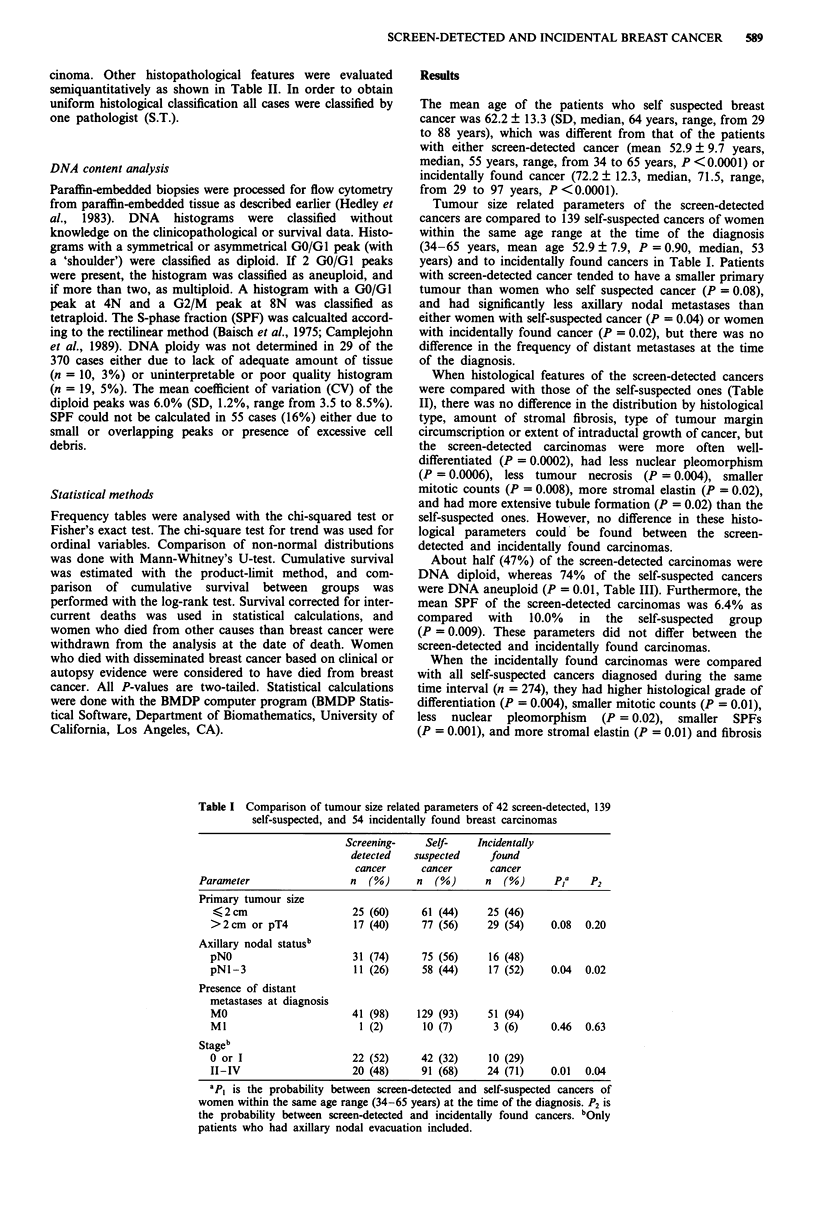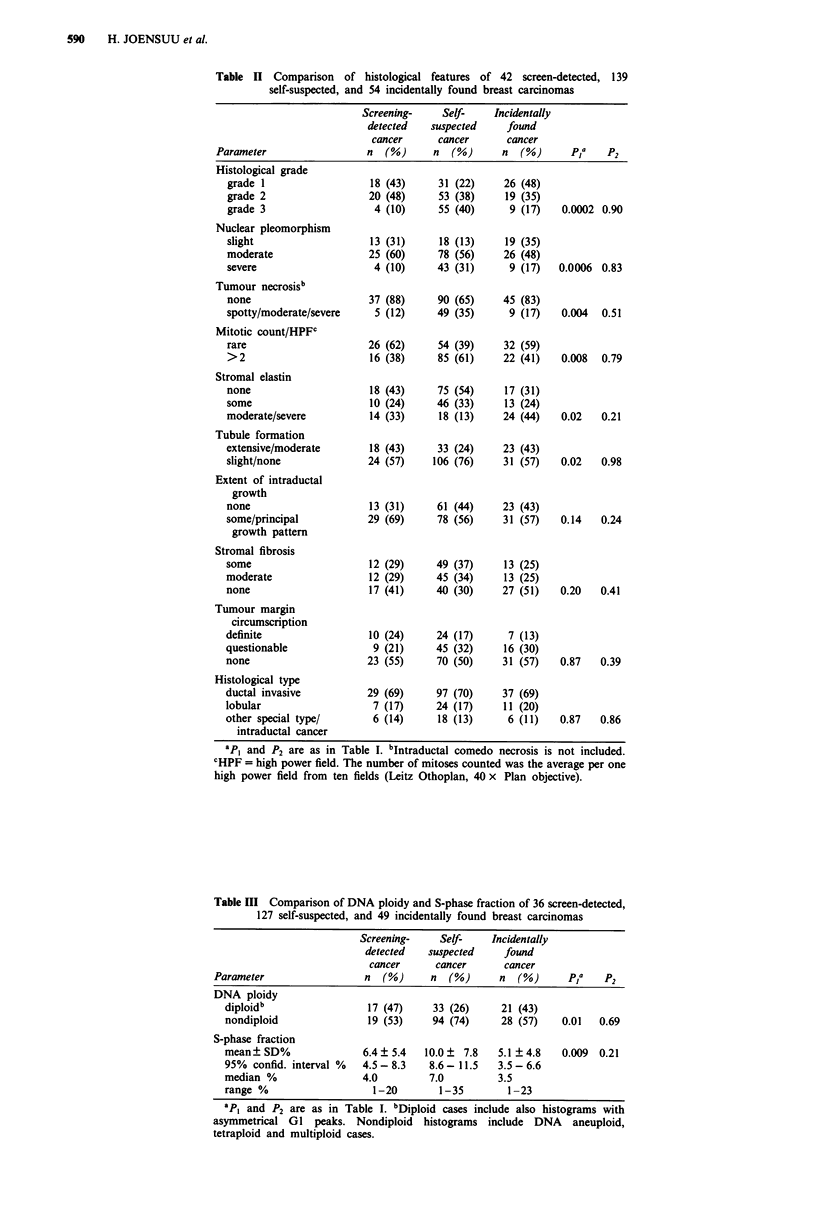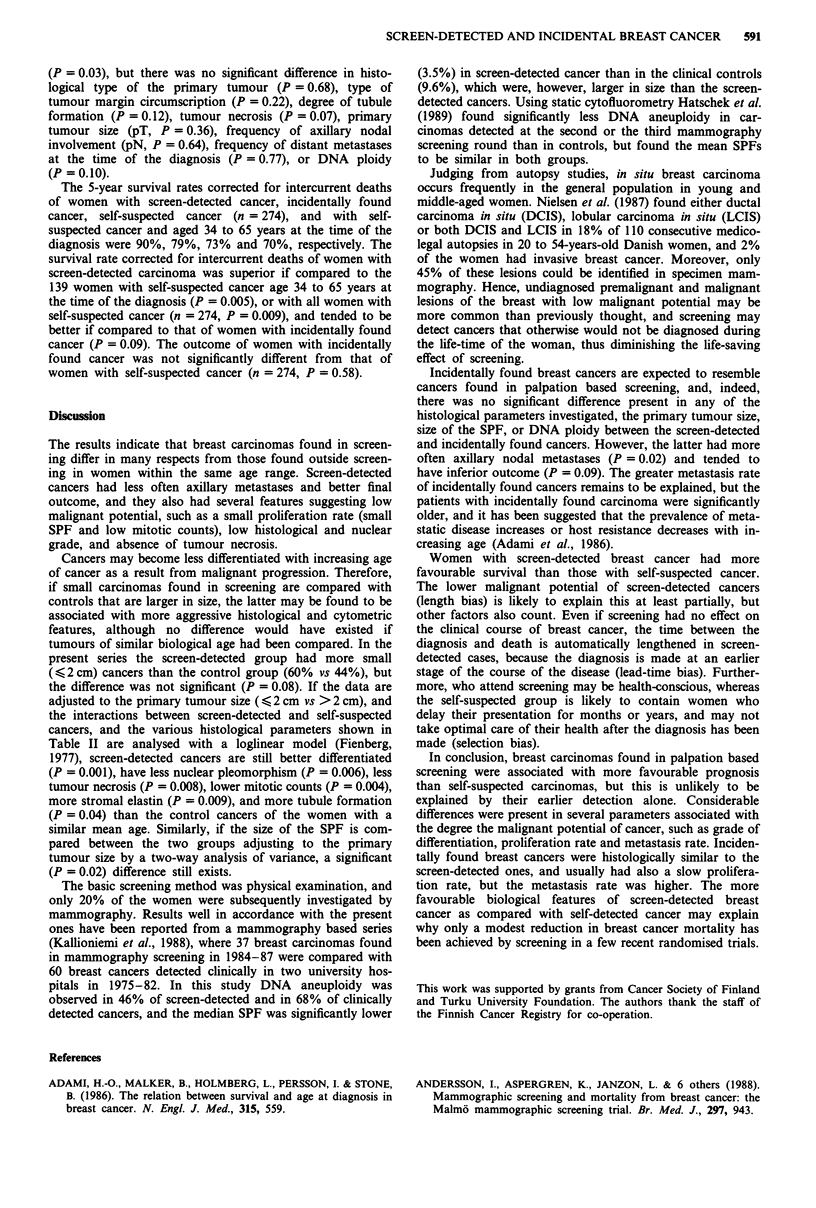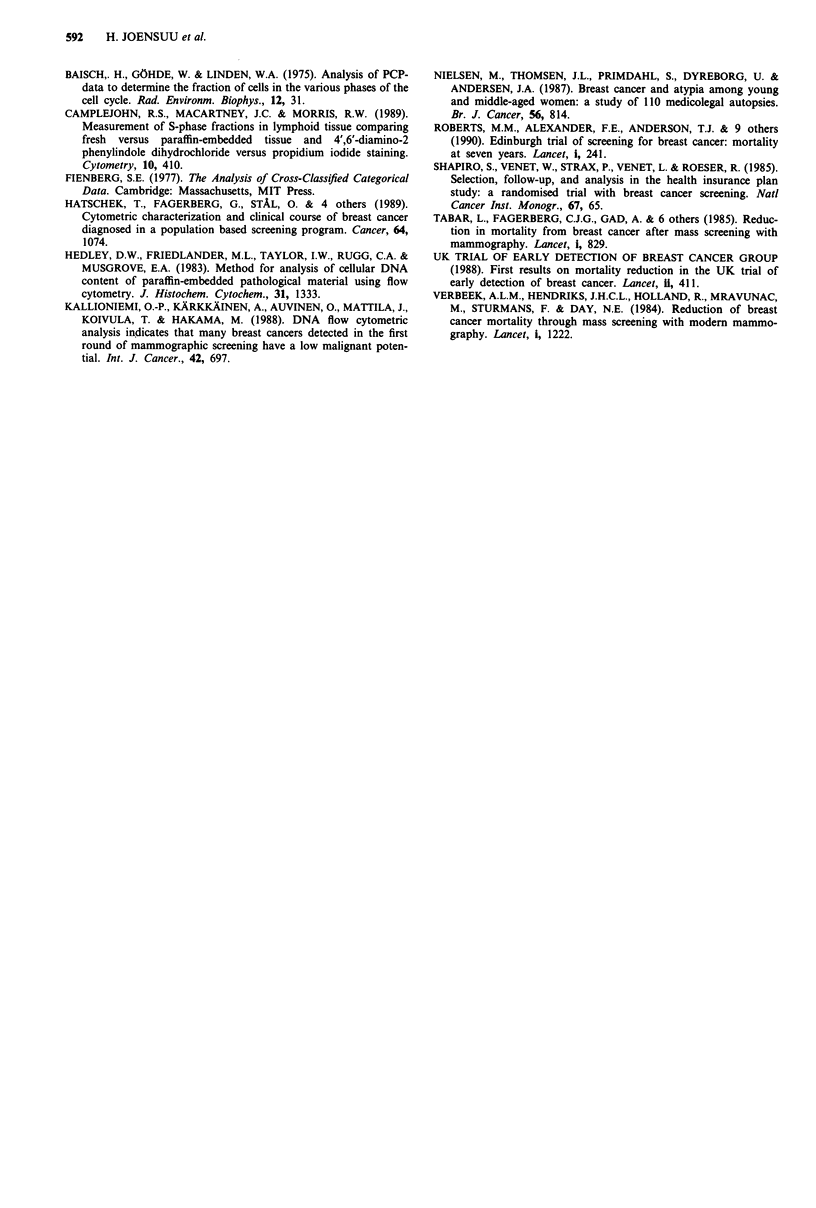# Histological features, DNA content and prognosis of breast carcinoma found incidentally or in screening.

**DOI:** 10.1038/bjc.1991.355

**Published:** 1991-09

**Authors:** H. Joensuu, S. Toikkanen, P. J. Klemi

**Affiliations:** Department of Radiotherapy, Turku University Central Hospital, Finland.

## Abstract

Histology features, the nuclear DNA content and prognosis of 42 female breast carcinomas found in a physical examination based screening, 54 breast cancers found incidentally by medical personnel, and 274 breast cancers first suspected by the patient were compared. There was no significant difference in the distribution by primary tumour size (P = 0.08) or histological type (P = 0.87) of breast cancer between the screen-detected and 139 self-suspected cancers of women with similar mean age and living at the same time in the same city, but the screen-detected carcinomas were better differentiated (P = 0.0002), and had less mitoses (P = 0.008), less tumour necrosis (P = 0.004) and DNA aneuploidy (P = 0.01), smaller S-phase fractions (P = 0.009), less axillary metastases (P = 0.04), and had better outcome (P = 0.005) than self-suspected carcinomas. These parameters did not differ significantly between the screen-detected and incidentally found cancers, but incidental cancers had more often axillary metastases (P = 0.02). The results indicate that screen-detected breast carcinomas have favourable biological features suggesting low degree of malignant potential.


Br. J. Cancer (1991), 64, 588-592 © Macmillan Press Ltd., 1991

### Histological features, DNA content and prognosis of breast carcinoma found incidentally or in screening

H. Joensuu[1], S. Toikkanen[3] & P.J. Klemi[2]

*The Departments of [1]Radiotherapy and Oncology, and [2]Pathology, Turku University Central Hospital, and Department of [3]Pathology, University of Turku, Turku, Finland.*

**Summary** Histology features, the nuclear DNA content and prognosis of 42 female breast carcinomas found in a physical examination based screening, 54 breast cancers found incidentally by medical personnel, and 274 breast cancers first suspected by the patient were compared. There was no significant difference in the distribution by primary tumour size ($P = 0.08$) or histological type ($P = 0.87$) of breast cancer between the screen-detected and 139 self-suspected cancers of women with similar mean age and living at the same time in the same city, but the screen-detected carcinomas were better differentiated ($P = 0.0002$), and had less mitoses ($P = 0.008$), less tumour necrosis ($P = 0.004$) and DNA aneuploidy ($P = 0.01$), smaller S-phase fractions ($P = 0.009$), less axillary metastases ($P = 0.04$), and had better outcome ($P = 0.005$) than self-suspected carcinomas. These parameters did not differ significantly between the screen-detected and incidentally found cancers, but incidental cancers had more often axillary metastases ($P = 0.02$). The results indicate that screen-detected breast carcinomas have favourable biological features suggesting low degree of malignant potential.

Breast carcinoma is usually first detected by the woman herself, who notices a lump in her breast. A few carcinomas are found incidentally by medical personnel, and an increasing number is found in screening programs. Carcinomas found in mammographic screening have been reported to be of lower stage than cancers found in the control group (Tabar *et al.*, 1985; Andersson *et al.*, 1988), and some trials have indicated that mammographic screening reduces mortality in breast cancer (Shapiro *et al.*, 1983; Verbeek *et al.*, 1984; Tabar *et al.*, 1985), whereas others have been less conclusive (Andersson *et al.*, 1988; UK Trial of Early Detection of Breast Cancer Group, 1988; Roberts *et al.*, 1990).

Breast cancer found in screening or incidentally during physical examination may form a special group of cancers with a slow progression rate. There are currently little data available to characterise screen-detected and incidentally found breast carcinomas, which would be of value in treatment planning. We have compared clinical presentation, histology, DNA ploidy and prognosis of breast carcinomas first suspected by the patient to those found incidentally by the medical personnel, and to those found in a palpation based mass screening.

### Materials and methods

### *Patients*

According to data from the Finnish Cancer Registry of Finland 404 cases of breast cancer were diagnosed in Turku in 1980 to 1984. After histological review, nine cases were either benign tumours or some other type of cancer than breast carcinoma. In two cases no biopsy had been taken and in one case it was taken at autopsy, and in 22 cases either an adequate histological sample or clinical data was lacking leaving 370 patients for analysis, which is 94.1% of all histologically diagnosed cases ($n = 393$) of breast carcinoma in the area. The median follow-up time was 6 years (range, from 4 to 9 years).

During 1980-84, 27,618 women were screened for cervical cancer in Turku, South-Western Finland. Screening was repeated with 5 years' intervals. In conjunction with this screening palpation of the breasts was performed by an experienced nurse, and subsequently suspicious or large breasts, and those otherwise difficult to examine were examined by mammography ($n = 5,481$, 20%).

According to data from hospital records, which were reviewed, 42 (11%) of the 370 cases of breast cancer were found in the palpation-based screening (referred to as 'screen-detected cancers'). All were found during the first round of screening. In addition, 54 cancers (15%) had been found incidentally during physical examination by physicians ($n = 49$) or nurses ($n = 5$, referred to as 'incidentally found cancers'). The rest of the tumours ($n = 274$, 74%) were first noticed or suspected by the patient (tumour in the breast, 87%; pain or abnormal sensations in the breast, 9%; discharge, 4%; dimpling of the skin, 4%; erythema or other skin disorder, 3%; symptom or sign not known, 5%, referred to as 'self-suspected cancers').

Clinical staging was done according to the postsurgical UICC TNM classification (1987). The patients were usually treated either with radical mastectomy or with mastectomy and evacuation of the axillary lymph nodes (88%), but in 21 self-suspected cases and in 18 incidentally found cases simple mastectomy had been performed. Two patients had partial mastectomy only (both in the incidentally found group), and two had biopsy only (both in the self-suspected group). Post-operative radiotherapy was given to 198 patients (54%, to 155 in the self-suspected group, 28 in the screen-detected group, and to 15 in the incidentally found group). Adjuvant cytostatic therapy with cyclophosphamide, methotrexate and 5-fluorouracil (CMF) was given to 10 (3%) patients (to one screen-detected and nine self-suspected) and adjuvant tamoxifen to 29 (8%) patients (one screen-detected, five incidentally found and 23 self-suspected).

### *Histology*

New haematoxylin-eosin and van Gieson stained slides were prepared from each tissue block, and the original van Gieson stained slides were reviewed. The histological typing and grading of the tumours was done slightly modifying the WHO classification, and the tumours were subsequently classified into four types: (1) infiltrating ductal carcinomas NOS (not otherwise specified, includes apocrine, mixed mucinous, and atypical medullary types), (2) infiltrating lobular carcinoma (includes variants), (3) other special types (includes tubular, medullary, cribriform, papillary, metaplastic and pure mucinous carcinomas), and (4) intraductal car-

Correspondence: H. Joensuu.
Received 30 November 1990, and in revised form 19 April 1991.

cinoma. Other histopathological features were evaluated semiquantitatively as shown in Table II. In order to obtain uniform histological classification all cases were classified by one pathologist (S.T.).

### *DNA content analysis*

Paraffin-embedded biopsies were processed for flow cytometry from paraffin-embedded tissue as described earlier (Hedley *et al.*, 1983). DNA histograms were classified without knowledge on the clinicopathological or survival data. Histograms with a symmetrical or asymmetrical G0/G1 peak (with a 'shoulder') were classified as diploid. If 2 G0/G1 peaks were present, the histogram was classified as aneuploid, and if more than two, as multiploid. A histogram with a G0/G1 peak at 4N and a G2/M peak at 8N was classified as tetraploid. The S-phase fraction (SPF) was calcualted according to the rectilinear method (Baisch *et al.*, 1975; Camplejohn *et al.*, 1989). DNA ploidy was not determined in 29 of the 370 cases either due to lack of adequate amount of tissue ($n$ = 10, 3%) or uninterpretable or poor quality histogram ($n$ = 19, 5%). The mean coefficient of variation (CV) of the diploid peaks was 6.0% (SD, 1.2%, range from 3.5 to 8.5%). SPF could not be calculated in 55 cases (16%) either due to small or overlapping peaks or presence of excessive cell debris.

### *Statistical methods*

Frequency tables were analysed with the chi-squared test or Fisher's exact test. The chi-square test for trend was used for ordinal variables. Comparison of non-normal distributions was done with Mann-Whitney's U-test. Cumulative survival was estimated with the product-limit method, and comparison of cumulative survival between groups was performed with the log-rank test. Survival corrected for intercurrent deaths was used in statistical calculations, and women who died from other causes than breast cancer were withdrawn from the analysis at the date of death. Women who died with disseminated breast cancer based on clinical or autopsy evidence were considered to have died from breast cancer. All $P$-values are two-tailed. Statistical calculations were done with the BMDP computer program (BMDP Statistical Software, Department of Biomathematics, University of California, Los Angeles, CA).

### Results

The mean age of the patients who self suspected breast cancer was 62.2 ± 13.3 (SD, median, 64 years, range, from 29 to 88 years), which was different from that of the patients with either screen-detected cancer (mean 52.9 ± 9.7 years, median, 55 years, range, from 34 to 65 years, $P < 0.0001$) or incidentally found cancer (72.2 ± 12.3, median, 71.5, range, from 29 to 97 years, $P < 0.0001$).

Tumour size related parameters of the screen-detected cancers are compared to 139 self-suspected cancers of women within the same age range at the time of the diagnosis (34–65 years, mean age 52.9 ± 7.9, $P = 0.90$, median, 53 years) and to incidentally found cancers in Table I. Patients with screen-detected cancer tended to have a smaller primary tumour than women who self suspected cancer ($P = 0.08$), and had significantly less axillary nodal metastases than either women with self-suspected cancer ($P = 0.04$) or women with incidentally found cancer ($P = 0.02$), but there was no difference in the frequency of distant metastases at the time of the diagnosis.

When histological features of the screen-detected cancers were compared with those of the self-suspected ones (Table II), there was no difference in the distribution by histological type, amount of stromal fibrosis, type of tumour margin circumscription or extent of intraductal growth of cancer, but the screen-detected carcinomas were more often well-differentiated ($P = 0.0002$), had less nuclear pleomorphism ($P = 0.0006$), less tumour necrosis ($P = 0.004$), smaller mitotic counts ($P = 0.008$), more stromal elastin ($P = 0.02$), and had more extensive tubule formation ($P = 0.02$) than the self-suspected ones. However, no difference in these histological parameters could be found between the screen-detected and incidentally found carcinomas.

About half (47%) of the screen-detected carcinomas were DNA diploid, whereas 74% of the self-suspected cancers were DNA aneuploid ($P = 0.01$, Table III). Furthermore, the mean SPF of the screen-detected carcinomas was 6.4% as compared with 10.0% in the self-suspected group ($P = 0.009$). These parameters did not differ between the screen-detected and incidentally found carcinomas.

When the incidentally found carcinomas were compared with all self-suspected cancers diagnosed during the same time interval ($n$ = 274), they had higher histological grade of differentiation ($P = 0.004$), smaller mitotic counts ($P = 0.01$), less nuclear pleomorphism ($P = 0.02$), smaller SPFs ($P = 0.001$), and more stromal elastin ($P = 0.01$) and fibrosis

**Table I** Comparison of tumour size related parameters of 42 screen-detected, 139 self-suspected, and 54 incidentally found breast carcinomas

| *Parameter* | *Screening-detected cancer* n (%) | *Self-suspected cancer* n (%) | *Incidentally found cancer* n (%) | $P_1^a$ | $P_2$ |
|---|---|---|---|---|---|
| Primary tumour size | | | | | |
| ≤2 cm | 25 (60) | 61 (44) | 25 (46) | | |
| >2 cm or pT4 | 17 (40) | 77 (56) | 29 (54) | 0.08 | 0.20 |
| Axillary nodal status[b] | | | | | |
| pN0 | 31 (74) | 75 (56) | 16 (48) | | |
| pN1-3 | 11 (26) | 58 (44) | 17 (52) | 0.04 | 0.02 |
| Presence of distant metastases at diagnosis | | | | | |
| M0 | 41 (98) | 129 (93) | 51 (94) | | |
| M1 | 1 (2) | 10 (7) | 3 (6) | 0.46 | 0.63 |
| Stage[b] | | | | | |
| 0 or I | 22 (52) | 42 (32) | 10 (29) | | |
| II-IV | 20 (48) | 91 (68) | 24 (71) | 0.01 | 0.04 |

[a]$P_1$ is the probability between screen-detected and self-suspected cancers of women within the same age range (34–65 years) at the time of the diagnosis. $P_2$ is the probability between screen-detected and incidentally found cancers. [b]Only patients who had axillary nodal evacuation included.

**Table II** Comparison of histological features of 42 screen-detected, 139 self-suspected, and 54 incidentally found breast carcinomas

| *Parameter* | *Screening-detected cancer n (%)* | *Self-suspected cancer n (%)* | *Incidentally found cancer n (%)* | $P_1^a$ | $P_2$ |
|---|---|---|---|---|---|
| Histological grade | | | | | |
| grade 1 | 18 (43) | 31 (22) | 26 (48) | | |
| grade 2 | 20 (48) | 53 (38) | 19 (35) | | |
| grade 3 | 4 (10) | 55 (40) | 9 (17) | 0.0002 | 0.90 |
| Nuclear pleomorphism | | | | | |
| slight | 13 (31) | 18 (13) | 19 (35) | | |
| moderate | 25 (60) | 78 (56) | 26 (48) | | |
| severe | 4 (10) | 43 (31) | 9 (17) | 0.0006 | 0.83 |
| Tumour necrosis[b] | | | | | |
| none | 37 (88) | 90 (65) | 45 (83) | | |
| spotty/moderate/severe | 5 (12) | 49 (35) | 9 (17) | 0.004 | 0.51 |
| Mitotic count/HPF[c] | | | | | |
| rare | 26 (62) | 54 (39) | 32 (59) | | |
| >2 | 16 (38) | 85 (61) | 22 (41) | 0.008 | 0.79 |
| Stromal elastin | | | | | |
| none | 18 (43) | 75 (54) | 17 (31) | | |
| some | 10 (24) | 46 (33) | 13 (24) | | |
| moderate/severe | 14 (33) | 18 (13) | 24 (44) | 0.02 | 0.21 |
| Tubule formation | | | | | |
| extensive/moderate | 18 (43) | 33 (24) | 23 (43) | | |
| slight/none | 24 (57) | 106 (76) | 31 (57) | 0.02 | 0.98 |
| Extent of intraductal growth | | | | | |
| none | 13 (31) | 61 (44) | 23 (43) | | |
| some/principal growth pattern | 29 (69) | 78 (56) | 31 (57) | 0.14 | 0.24 |
| Stromal fibrosis | | | | | |
| some | 12 (29) | 49 (37) | 13 (25) | | |
| moderate | 12 (29) | 45 (34) | 13 (25) | | |
| none | 17 (41) | 40 (30) | 27 (51) | 0.20 | 0.41 |
| Tumour margin circumscription | | | | | |
| definite | 10 (24) | 24 (17) | 7 (13) | | |
| questionable | 9 (21) | 45 (32) | 16 (30) | | |
| none | 23 (55) | 70 (50) | 31 (57) | 0.87 | 0.39 |
| Histological type | | | | | |
| ductal invasive | 29 (69) | 97 (70) | 37 (69) | | |
| lobular | 7 (17) | 24 (17) | 11 (20) | | |
| other special type/ intraductal cancer | 6 (14) | 18 (13) | 6 (11) | 0.87 | 0.86 |

[a]$P_1$ and $P_2$ are as in Table I. [b]Intraductal comedo necrosis is not included. [c]HPF = high power field. The number of mitoses counted was the average per one high power field from ten fields (Leitz Othoplan, 40 × Plan objective).

**Table III** Comparison of DNA ploidy and S-phase fraction of 36 screen-detected, 127 self-suspected, and 49 incidentally found breast carcinomas

| *Parameter* | *Screening-detected cancer n (%)* | *Self-suspected cancer n (%)* | *Incidentally found cancer n (%)* | $P_1^a$ | $P_2$ |
|---|---|---|---|---|---|
| DNA ploidy | | | | | |
| diploid[b] | 17 (47) | 33 (26) | 21 (43) | | |
| nondiploid | 19 (53) | 94 (74) | 28 (57) | 0.01 | 0.69 |
| S-phase fraction | | | | | |
| mean ± SD% | 6.4 ± 5.4 | 10.0 ± 7.8 | 5.1 ± 4.8 | 0.009 | 0.21 |
| 95% confid. interval % | 4.5 – 8.3 | 8.6 – 11.5 | 3.5 – 6.6 | | |
| median % | 4.0 | 7.0 | 3.5 | | |
| range % | 1–20 | 1–35 | 1–23 | | |

[a]$P_1$ and $P_2$ are as in Table I. [b]Diploid cases include also histograms with asymmetrical G1 peaks. Nondiploid histograms include DNA aneuploid, tetraploid and multiploid cases.

($P = 0.03$), but there was no significant difference in histological type of the primary tumour ($P = 0.68$), type of tumour margin circumscription ($P = 0.22$), degree of tubule formation ($P = 0.12$), tumour necrosis ($P = 0.07$), primary tumour size (pT, $P = 0.36$), frequency of axillary nodal involvement (pN, $P = 0.64$), frequency of distant metastases at the time of the diagnosis ($P = 0.77$), or DNA ploidy ($P = 0.10$).

The 5-year survival rates corrected for intercurrent deaths of women with screen-detected cancer, incidentally found cancer, self-suspected cancer ($n = 274$), and with self-suspected cancer and aged 34 to 65 years at the time of the diagnosis were 90%, 79%, 73% and 70%, respectively. The survival rate corrected for intercurrent deaths of women with screen-detected carcinoma was superior if compared to the 139 women with self-suspected cancer age 34 to 65 years at the time of the diagnosis ($P = 0.005$), or with all women with self-suspected cancer ($n = 274$, $P = 0.009$), and tended to be better if compared to that of women with incidentally found cancer ($P = 0.09$). The outcome of women with incidentally found cancer was not significantly different from that of women with self-suspected cancer ($n = 274$, $P = 0.58$).

### Discussion

The results indicate that breast carcinomas found in screening differ in many respects from those found outside screening in women within the same age range. Screen-detected cancers had less often axillary metastases and better final outcome, and they also had several features suggesting low malignant potential, such as a small proliferation rate (small SPF and low mitotic counts), low histological and nuclear grade, and absence of tumour necrosis.

Cancers may become less differentiated with increasing age of cancer as a result from malignant progression. Therefore, if small carcinomas found in screening are compared with controls that are larger in size, the latter may be found to be associated with more aggressive histological and cytometric features, although no difference would have existed if tumours of similar biological age had been compared. In the present series the screen-detected group had more small ($\leqslant 2$ cm) cancers than the control group (60% *vs* 44%), but the difference was not significant ($P = 0.08$). If the data are adjusted to the primary tumour size ($\leqslant 2$ cm *vs* $> 2$ cm), and the interactions between screen-detected and self-suspected cancers, and the various histological parameters shown in Table II are analysed with a loglinear model (Fienberg, 1977), screen-detected cancers are still better differentiated ($P = 0.001$), have less nuclear pleomorphism ($P = 0.006$), less tumour necrosis ($P = 0.008$), lower mitotic counts ($P = 0.004$), more stromal elastin ($P = 0.009$), and more tubule formation ($P = 0.04$) than the control cancers of the women with a similar mean age. Similarly, if the size of the SPF is compared between the two groups adjusting to the primary tumour size by a two-way analysis of variance, a significant ($P = 0.02$) difference still exists.

The basic screening method was physical examination, and only 20% of the women were subsequently investigated by mammography. Results well in accordance with the present ones have been reported from a mammography based series (Kallioniemi *et al.*, 1988), where 37 breast carcinomas found in mammography screening in 1984–87 were compared with 60 breast cancers detected clinically in two university hospitals in 1975–82. In this study DNA aneuploidy was observed in 46% of screen-detected and in 68% of clinically detected cancers, and the median SPF was significantly lower (3.5%) in screen-detected cancer than in the clinical controls (9.6%), which were, however, larger in size than the screen-detected cancers. Using static cytofluorometry Hatschek *et al.* (1989) found significantly less DNA aneuploidy in carcinomas detected at the second or the third mammography screening round than in controls, but found the mean SPFs to be similar in both groups.

Judging from autopsy studies, *in situ* breast carcinoma occurs frequently in the general population in young and middle-aged women. Nielsen *et al.* (1987) found either ductal carcinoma *in situ* (DCIS), lobular carcinoma *in situ* (LCIS) or both DCIS and LCIS in 18% of 110 consecutive medico-legal autopsies in 20 to 54-years-old Danish women, and 2% of the women had invasive breast cancer. Moreover, only 45% of these lesions could be identified in specimen mammography. Hence, undiagnosed premalignant and malignant lesions of the breast with low malignant potential may be more common than previously thought, and screening may detect cancers that otherwise would not be diagnosed during the life-time of the woman, thus diminishing the life-saving effect of screening.

Incidentally found breast cancers are expected to resemble cancers found in palpation based screening, and, indeed, there was no significant difference present in any of the histological parameters investigated, the primary tumour size, size of the SPF, or DNA ploidy between the screen-detected and incidentally found cancers. However, the latter had more often axillary nodal metastases ($P = 0.02$) and tended to have inferior outcome ($P = 0.09$). The greater metastasis rate of incidentally found cancers remains to be explained, but the patients with incidentally found carcinoma were significantly older, and it has been suggested that the prevalence of metastatic disease increases or host resistance decreases with increasing age (Adami *et al.*, 1986).

Women with screen-detected breast cancer had more favourable survival than those with self-suspected cancer. The lower malignant potential of screen-detected cancers (length bias) is likely to explain this at least partially, but other factors also count. Even if screening had no effect on the clinical course of breast cancer, the time between the diagnosis and death is automatically lengthened in screen-detected cases, because the diagnosis is made at an earlier stage of the course of the disease (lead-time bias). Furthermore, who attend screening may be health-conscious, whereas the self-suspected group is likely to contain women who delay their presentation for months or years, and may not take optimal care of their health after the diagnosis has been made (selection bias).

In conclusion, breast carcinomas found in palpation based screening were associated with more favourable prognosis than self-suspected carcinomas, but this is unlikely to be explained by their earlier detection alone. Considerable differences were present in several parameters associated with the degree the malignant potential of cancer, such as grade of differentiation, proliferation rate and metastasis rate. Incidentally found breast cancers were histologically similar to the screen-detected ones, and usually had also a slow proliferation rate, but the metastasis rate was higher. The more favourable biological features of screen-detected breast cancer as compared with self-detected cancer may explain why only a modest reduction in breast cancer mortality has been achieved by screening in a few recent randomised trials.

This work was supported by grants from Cancer Society of Finland and Turku University Foundation. The authors thank the staff of the Finnish Cancer Registry for co-operation.

### References

ADAMI, H.-O., MALKER, B., HOLMBERG, L., PERSSON, I. & STONE, B. (1986). The relation between survival and age at diagnosis in breast cancer. *N. Engl. J. Med.*, **315**, 559.

ANDERSSON, I., ASPERGREN, K., JANZON, L. & 6 others (1988). Mammographic screening and mortality from breast cancer: the Malmö mammographic screening trial. *Br. Med. J.*, **297**, 943.

BAISCH,. H., GÖHDE, W. & LINDEN, W.A. (1975). Analysis of PCP-data to determine the fraction of cells in the various phases of the cell cycle. *Rad. Environm. Biophys.*, **12**, 31.

CAMPLEJOHN, R.S., MACARTNEY, J.C. & MORRIS, R.W. (1989). Measurement of S-phase fractions in lymphoid tissue comparing fresh versus paraffin-embedded tissue and 4',6'-diamino-2 phenylindole dihydrochloride versus propidium iodide staining. *Cytometry*, **10**, 410.

FIENBERG, S.E. (1977). *The Analysis of Cross-Classified Categorical Data*. Cambridge: Massachusetts, MIT Press.

HATSCHEK, T., FAGERBERG, G., STÅL, O. & 4 others (1989). Cytometric characterization and clinical course of breast cancer diagnosed in a population based screening program. *Cancer*, **64**, 1074.

HEDLEY, D.W., FRIEDLANDER, M.L., TAYLOR, I.W., RUGG, C.A. & MUSGROVE, E.A. (1983). Method for analysis of cellular DNA content of paraffin-embedded pathological material using flow cytometry. *J. Histochem. Cytochem.*, **31**, 1333.

KALLIONIEMI, O.-P., KÄRKKÄINEN, A., AUVINEN, O., MATTILA, J., KOIVULA, T. & HAKAMA, M. (1988). DNA flow cytometric analysis indicates that many breast cancers detected in the first round of mammographic screening have a low malignant potential. *Int. J. Cancer.*, **42**, 697.

NIELSEN, M., THOMSEN, J.L., PRIMDAHL, S., DYREBORG, U. & ANDERSEN, J.A. (1987). Breast cancer and atypia among young and middle-aged women: a study of 110 medicolegal autopsies. *Br. J. Cancer*, **56**, 814.

ROBERTS, M.M., ALEXANDER, F.E., ANDERSON, T.J. & 9 others (1990). Edinburgh trial of screening for breast cancer: mortality at seven years. *Lancet*, **i**, 241.

SHAPIRO, S., VENET, W., STRAX, P., VENET, L. & ROESER, R. (1985). Selection, follow-up, and analysis in the health insurance plan study: a randomised trial with breast cancer screening. *Natl Cancer Inst. Monogr.*, **67**, 65.

TABAR, L., FAGERBERG, C.J.G., GAD, A. & 6 others (1985). Reduction in mortality from breast cancer after mass screening with mammography. *Lancet*, **i**, 829.

UK TRIAL OF EARLY DETECTION OF BREAST CANCER GROUP (1988). First results on mortality reduction in the UK trial of early detection of breast cancer. *Lancet*, **ii**, 411.

VERBEEK, A.L.M., HENDRIKS, J.H.C.L., HOLLAND, R., MRAVUNAC, M., STURMANS, F. & DAY, N.E. (1984). Reduction of breast cancer mortality through mass screening with modern mammography. *Lancet*, **i**, 1222.